# Durable hydrogen evolution from water driven by sunlight using (Ag,Cu)GaSe_2_ photocathodes modified with CdS and CuGa_3_Se_5_ †
†Electronic supplementary information (ESI) available. See DOI: 10.1039/c4sc02346c



**DOI:** 10.1039/c4sc02346c

**Published:** 2014-09-04

**Authors:** Li Zhang, Tsutomu Minegishi, Mamiko Nakabayashi, Yohichi Suzuki, Kazuhiko Seki, Naoya Shibata, Jun Kubota, Kazunari Domen

**Affiliations:** a Department of Chemical System Engineering , The University of Tokyo , 7-3-1 Hongo , Bunkyo-ku , Tokyo 113-8656 , Japan . Email: domen@chemsys.t.u-tokyo.ac.jp ; Fax: +81-3-5841-8838 ; Tel: +81-3-5841-1652; b Institute of Engineering Innovation , The University of Tokyo , 2-11-16 Yayoi , Bunkyo-ku , Tokyo 113-8656 , Japan; c NRI , National Institute of Advanced Industrial Science and Technology (AIST) , AIST Tsukuba Central 5, Higashi 1-1-1 , Tsukuba , Ibaraki 305-8565 , Japan

## Abstract

The photoelectrochemical properties of (Ag,Cu)GaSe_2_ (ACGSe) modified by deposition of CdS and CuGa_3_Se_5_ layers were investigated. The CdS and CuGa_3_Se_5_ layers formed p–n junctions with an appropriate band diagram on the surface of the electrode and they clearly increased the cathodic photocurrent and onset potential. The Pt, CdS, and CuGa_3_Se_5_ modified ACGSe (Pt/CdS/CuGa_3_Se_5_/ACGSe) with an appropriate thickness of CuGa_3_Se_5_ layers (*ca.* 100 nm) showed a cathodic photocurrent of 8.79 mA cm^–2^ at 0 V_RHE_ and an onset potential of 0.62 V_RHE_ (defined as cathodic photocurrent of 1.0 mA cm^–2^) under simulated sunlight irradiation in 0.1 M Na_2_HPO_4_ (pH 10). Pt/CdS/CuGa_3_Se_5_/ACGSe showed durable cathodic current under the observed reaction conditions and hydrogen was evolved for about 20 days.

## Abstract 2

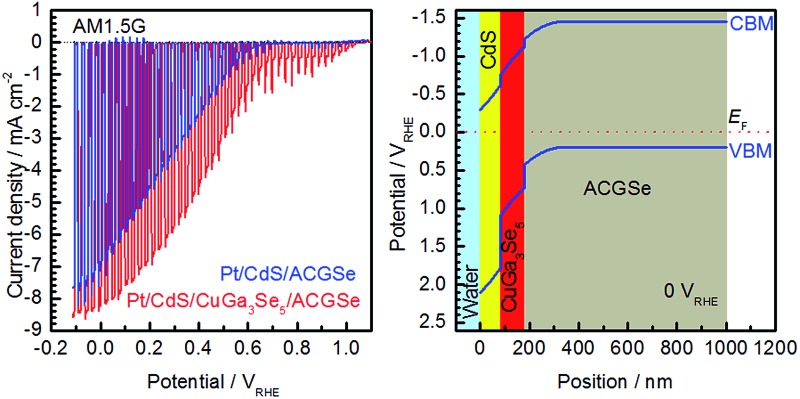
The multilayer structure enhances the hydrogen evolution from water under simulated sunlight.

## Introduction

Sustainable energy supply is one of the greatest challenges facing human society today.^1,2^ Global energy consumption was equivalent to *ca.* 1.5 × 10^1^ TW in 2008, and is predicted to double by 2050 and triple by 2100.^3^ Utilization of solar energy appears to be our best option for addressing the decreased availability of finite fossil fuels and the increase of anthropogenic carbon dioxide emissions, because the solar energy reaching the Earth (1.3 × 10^5^ TW) is roughly four orders of magnitude greater than the global energy consumption.^4^

Solar energy generation through solar cells is one fascinating way to harvest energy from the sun. However, solar cells can generate electricity only during daytime and only on sunny days, and they should be coupled with efficient energy storage technology to overcome the diurnal intermittency of sunlight.^5^ Solar-driven water splitting for hydrogen production is potentially one of the most sustainable and efficient energy storage technologies.^4,6^ Storable and renewable hydrogen produced by solar-driven water splitting can be used directly to obtain mechanical energy using a combustion engine or be converted into electricity using a fuel cell as a clean fuel, whenever and wherever necessary.

Photoelectrochemical (PEC) water splitting, which integrates sunlight absorption and water electrolysis into a single semiconductor electrode, presents an attractive and efficient solution for solar-driven hydrogen production.^7–9^ The search for a single-photoelectrode PEC cell capable of bias-free overall water splitting with sufficient efficiency and adequate stability has proven to be quite difficult.^10^ A dual-photoelectrode PEC cell that consists of a photoanode and a photocathode with overlapped band gaps covering the oxygen evolution and hydrogen evolution potentials in side-by-side or top-to-bottom configurations, has been proposed as one of the most effective solutions with the potential to achieve solar-to-hydrogen (STH) conversion efficiencies of larger than 10% for large-scale applications.^10–12^

To drive efficient overall water splitting on a dual-photoelectrode PEC cell, a sufficiently large photocurrent at the intersection of the photocurrent–potential curves of the respective electrodes is necessary. Lower and higher onset potentials of the photocurrents from the photoanode and photocathode, respectively, are required in order to achieve efficient water splitting. Extensive research efforts have been devoted to photoanodes based on metal oxides (TiO_2_,^13^ α-Fe_2_O_3_,^14,15^ BiVO_4_,^16,17^ WO_3_,^18,19^ and ZnO^20^), metal (oxy)nitrides (TaON,^21^ Ta_3_N_5_,^22,23^ BaTiO_2_N,^24^ and LaTiO_2_N^25^), and n-type Si.^26,27^ Relatively fewer studies have focused on photocathodes.^28–33^ Single-crystalline p-type phosphides (InP^28^ and GaInP_2_ (ref. 29)) have been reported to be highly efficient photocathodes for hydrogen evolution. As Earth-abundant and inexpensive semiconductor materials, p-type Cu_2_O^30,31^ and p-type Si^32,33^ have been investigated as photocathodes for hydrogen evolution.

As one category of efficient sunlight-absorbing materials, I–III–VI_2_ chalcogenides (I = Cu; III = In, Ga; VI = S, Se) have emerged as a leading class of thin film solar cell materials, due to their tunable band gap (*ca.* 1.0–2.4 eV), high photo-absorption coefficients (*ca.* 10^5^ cm^–1^) and usability in the polycrystalline state.^34–37^ All of these beneficial properties have encouraged researchers to study the application of I–III–VI_2_ chalcogenides as photoelectrodes for PEC water splitting. Among the materials tested, CuGaSe_2_ (CGSe), with a direct band gap of 1.65 eV, is one of the more promising candidates as a photocathode for hydrogen evolution,^38–40^ because it can efficiently utilize a large portion of visible sunlight (absorption edge *ca.* 750 nm). Assuming an incident-photon-to-current-conversion efficiency (IPCE) of 100%, a CGSe photocathode can generate a photocurrent of 24 mA cm^–2^ above the absorption edge against AM 1.5G light (ASTM G173-03).^41^

Marsen *et al.* reported that a CGSe photocathode worked stably for hydrogen evolution in a strongly acidic electrolyte (0.5 M H_2_SO_4_), and they obtained a saturated photocurrent of 10.6 mA cm^–2^ at –0.9 V_SCE_ (*i.e.*, –0.66 V_RHE_) under simulated sunlight, although the onset potential was relatively low (*ca.* 0.136 V_RHE_).^38^ The low onset potential of CGSe photocathodes is due to its valence-band maximum (VBM) position, which is too shallow compared to the oxygen evolution potential in PEC water splitting. For Cu-based chalcogenide photocathodes, surface modification using a thin layer of n-type CdS to form a p–n heterojunction is an effective way to obtain higher onset potentials and cathodic photocurrents.^39,40,42–45^ Yokoyama *et al.* reported that CdS modification of Cu(In,Ga)Se_2_ and Cu_2_ZnSnS_4_ photocathodes clearly increased the photocurrents and onset potentials for hydrogen evolution.^42,43^ Ikeda *et al.* demonstrated an appreciable enhancement of the PEC response on a CuInS_2_ photocathode by the introduction of a thin CdS layer.^44,45^ Moriya *et al.* recently reported that a CGSe photocathode modified with CdS and Pt showed a marked increase in photocurrent and onset potential, owing to the enhanced charge separation by the formation of a p–n heterojunction, where Pt/CdS/CGSe reached a hypothetical half-cell solar-to-hydrogen efficiency (HC-STH) of 0.83% at 0.2 V_RHE_.^39^ More recently, the authors have reported an improvement of the CGSe electrodes by the partial substitution of Cu for Ag.^46^ The resulting (Ag,Cu)GaSe_2_ (ACGSe) electrodes modified with CdS and Pt showed an enhanced HC-STH (1.22% at 0.3 V_RHE_), since ACGSe has a larger grain size and a more positive VBM potential than CGSe. However, the measured efficiency of the Pt/CdS/ACGSe photocathode was still lower than that expected on the basis of the band gap of ACGSe (*ca.* 1.65 eV) and its flat-band potential. One possible reason for this might be a large amount of carrier recombination at the CdS/ACGSe interface due to the high defect density resulting from the large lattice mismatch, in addition to the unfavorably large band offset, as is commonly encountered in the case of CdS/CGSe interfaces in solar cells.^47,48^

Introducing a surface modification layer to form more desirable band alignments for the CdS/ACGSe heterojunction might help to reduce interfacial recombination, and thus enhance hydrogen evolution on the Pt/CdS/ACGSe photocathode. The ordered defect compound (ODC) of CGSe, CuGa_3_Se_5_, has emerged as a potential candidate for this purpose, owing to the fact that its band gap (*ca.* 1.85 eV) is larger than that of CGSe (or ACGSe) due to deepening of the VBM potential,^49,50^ and it is structurally similar to CGSe (or ACGSe) except for the periodic presence of (2V_Cu_^–^ + Ga_Cu_^2+^) defect pairs (*i.e.*, a single defect pair for each 5 units of CGSe).^51^ Rusu *et al.* studied the electronic properties of ODCs of CGSe, namely CuGa_3_Se_5_ and CuGa_5_Se_8_, and showed that they had deeper VBM positions than CGSe.^49^ Kim *et al.* investigated the structural and PEC properties of CuGa_3_Se_5_ (ref. 50 and 52) and found that CuGa_3_Se_5_ had p-type conductivity and a deeper VBM position than CGSe. After Pt modification, they observed a high onset potential (*ca.* 1.1 V_RHE_) on the CuGa_3_Se_5_ photocathode under simulated sunlight. However, the HC-STH for both Pt/CuGa_3_Se_5_ and Pt/ZnS/H_2_:CuGa_3_Se_5_ was less than 0.4%, presumably owing to the very small grain size in CuGa_3_Se_5_, which made it difficult to form a p–n junction because of strong Zn diffusion across the grain boundaries.

In the present study, the PEC and structural properties of CuGa_3_Se_5_-modified ACGSe (CuGa_3_Se_5_/ACGSe) thin film photocathodes decorated with CdS and Pt were investigated. It was found that Pt- and CdS-modified CuGa_3_Se_5_/ACGSe (Pt/CdS/CuGa_3_Se_5_/ACGSe) photocathodes showed a significantly higher photocurrent and onset potential than Pt/CdS/ACGSe photocathodes.

## Experimental

### Preparation of the CuGa_3_Se_5_/ACGSe thin films

CuGa_3_Se_5_/ACGSe thin films were prepared on Mo/Ti/SLG (SLG: soda lime glass) substrates by vacuum co-evaporation using a molecular beam epitaxy (MBE) system. The Mo/Ti/SLG substrates were prepared by radio frequency (RF) magnetron sputtering (see ESI†). Knudsen cells loaded with high-purity elemental sources of Ag (6 N), Cu (5 N), Ga (6 N) and Se (6 N) were placed in the vacuum chamber of the MBE system having a base pressure of *ca.* 1 × 10^–6^ Pa. Each of these cells consisted of a crucible, heating filaments, thermocouple, shutter and cooling line. The deposition rates for Ag, Cu, Ga and Se were monitored by an *in situ* quartz crystal microbalance (QCM) system and controlled by adjusting the source temperature. ACGSe films with a Ag/(Cu + Ag) ratio of *ca.* 5% were prepared by a two-step deposition method,^39,46^ in which the ACGSe thin films were first deposited at a relatively low substrate temperature of 450 °C for 10 min, followed by deposition at 550 °C for 90 min. The deposition rates for Ag, Cu and Ga were 0.03, 0.28 and 0.06 Å s^–1^ with a fluctuation of ±0.01 Å s^–1^ for each, resulting in ACGSe films with a Ag/(Cu + Ag) ratio of *ca.* 5% and a slight group I deficiency (Cu + Ag)/Ga of *ca.* 0.85. The deposition rate for Se was fixed at 8.00 ± 1.00 Å s^–1^ to ensure Se-rich conditions. For the CuGa_3_Se_5_ modified samples, CuGa_3_Se_5_ was deposited onto the grown ACGSe at 550 °C for 10 to 20 min under H_2_-mediated conditions.^52^ Pure H_2_ gas (5 N) was continuously introduced into the vacuum chamber and maintained at a constant pressure of ca. 5.0 ×10^–3^ Pa by adjusting the needle valve. The deposition rates for Cu and Ga were 0.12 and 0.06 Å s^–1^ with a fluctuation of ±0.01 Å s^–1^ for both elements. The deposition rate for Se was 4.00 ± 1.00 Å s^–1^, with a Se/metal flux ratio of *ca.* 4. Before the fabrication of the electrodes, CdS layers were formed on the surface of prepared CuGa_3_Se_5_/ACGSe films by the chemical bath deposition (CBD) method (see ESI†).

### Photoelectrochemical measurements

The prepared CdS/CuGa_3_Se_5_/ACGSe thin film samples were fabricated into electrodes and then surface-modified with Pt as a hydrogen evolution catalyst by PEC deposition (see ESI†). PEC measurements were performed using a typical 3-electrode setup consisting of a specimen, a coiled Pt wire, and an Ag/AgCl electrode in a saturated aqueous KCl solution as the working, counter, and reference electrodes, respectively. The potential of the working electrode was controlled by a potentiostat (Hokuto Denko, HSV-100). An aqueous solution of 0.1 M Na_2_HPO_4_ (99%, Wako) with the pH adjusted to 10 by NaOH addition was employed as the electrolyte. All PEC measurements were conducted under an Ar-purged atmosphere.

Photocurrent densities were measured under simulated sunlight illumination using an AM 1.5G solar simulator (XES-40S2-CE, San-ei Electric), as shown in Fig. S1 in ESI.† The wavelength dependence of the IPCE was measured under monochromatic light from a Xe lamp (Max-301, Asahi Spectra) equipped with band-pass filters (full width at half maximum (FWHM) ≈ 10 nm) and an optical fiber. Monochromatic light intensities were measured using a calibrated Si photodiode (S2281-01, Hamamatsu).

## Results and discussion

### PEC properties of the Pt/CdS/CuGa_3_Se_5_/ACGSe photocathodes

**Fig. 1:**
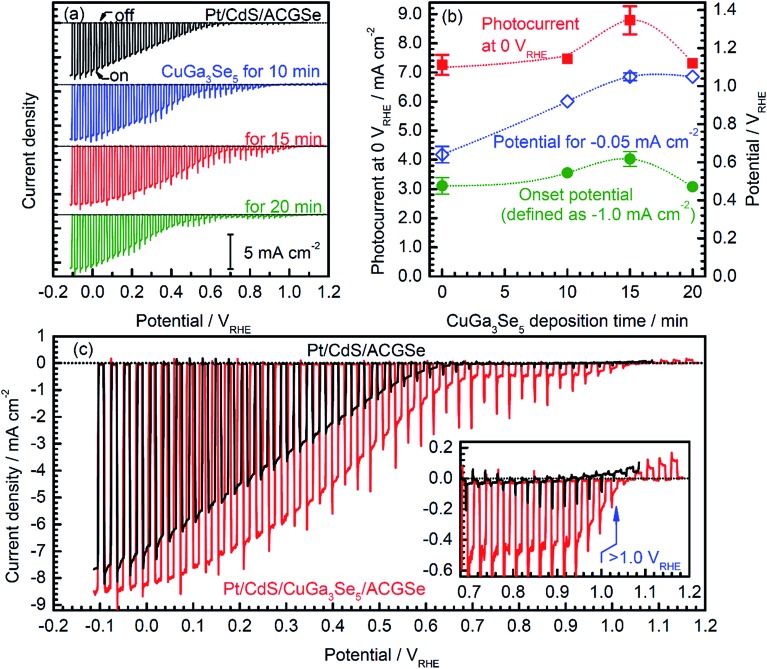
Current–potential (*I*–*E*) curves for Pt/CdS/CuGa_3_Se_5_/ACGSe with various CuGa_3_Se_5_ deposition times (a), plots of photocurrent density at 0 V_RHE_ and onset potential against CuGa_3_Se_5_ deposition time (b), and comparison of *I*–*E* curves for Pt/CdS/ACGSe and Pt/CdS/CuGa_3_Se_5_/ACGSe with CuGa_3_Se_5_ deposited for 15 min (c). A 0.1 M aqueous Na_2_HPO_4_ solution (adjusted to pH 10 by addition of NaOH) was employed as the electrolyte. An applied potential was swept in the positive direction at 5 mV s^–1^ under intermittent irradiation with simulated sunlight. Error bars show the standard deviation from the mean of the three samples.

Current–potential (*I*–*E*) curves for the Pt/CdS/CuGa_3_Se_5_/ACGSe electrodes with various deposition times for the CuGa_3_Se_5_ layer are shown in Fig. 1a, and the photocurrents at 0 V_RHE_ and onset potentials are plotted against the CuGa_3_Se_5_ deposition time in Fig. 1b. All of the Pt/CdS/CuGa_3_Se_5_/ACGSe samples showed an enhanced cathodic photocurrent compared to Pt/CdS/ACGSe, with a small photocurrent being observed for an applied potential of >0.6 V_RHE_. The Pt/CdS/CuGa_3_Se_5_/ACGSe electrode with CuGa_3_Se_5_ deposited for 15 min showed the highest cathodic photocurrent (8.79 mA cm^–2^ at 0 V_RHE_), and an onset potential of *ca.* 0.62 V_RHE_ (defined as a cathodic photocurrent of 1.0 mA cm^–2^) based on the averaged values from three samples (see Fig. S2 in ESI†). Compared with Pt/CdS/ACGSe, which showed a cathodic photocurrent of 7.26 mA cm^–2^ at 0 V_RHE_ and an onset potential of 0.48 V_RHE_, this Pt/CdS/CuGa_3_Se_5_/ACGSe sample showed a clear increase in photocurrent density over the entire potential range below the onset potential (see Fig. 1c), and, more importantly, a positive shift in the onset potential (*ca.* 140 mV). For Pt/CdS/CuGa_3_Se_5_/ACGSe, the maximum HC-STH was 1.81% at 0.36 V_RHE_ (see Fig. S3 in ESI†), while for Pt/CdS/ACGSe, it was 1.02% at 0.32 V_RHE_. It should be noted that the HC-STH for Pt/CdS/ACGSe is almost in agreement with the reported value.^46^ The observed small photocurrent for all of the Pt/CdS/CuGa_3_Se_5_/ACGSe samples at applied potential of >0.6 V_RHE_ was probably due to the photoexcited electrons generated in the CuGa_3_Se_5_ layer.^50^ The clear increase in onset potential on Pt/CdS/CuGa_3_Se_5_/ACGSe photocathodes can be ascribed to the contribution of the CuGa_3_Se_5_ layers. Possible mechanisms for the enhancement of the photocurrent and onset potential will be discussed below.

**Fig. 2:**
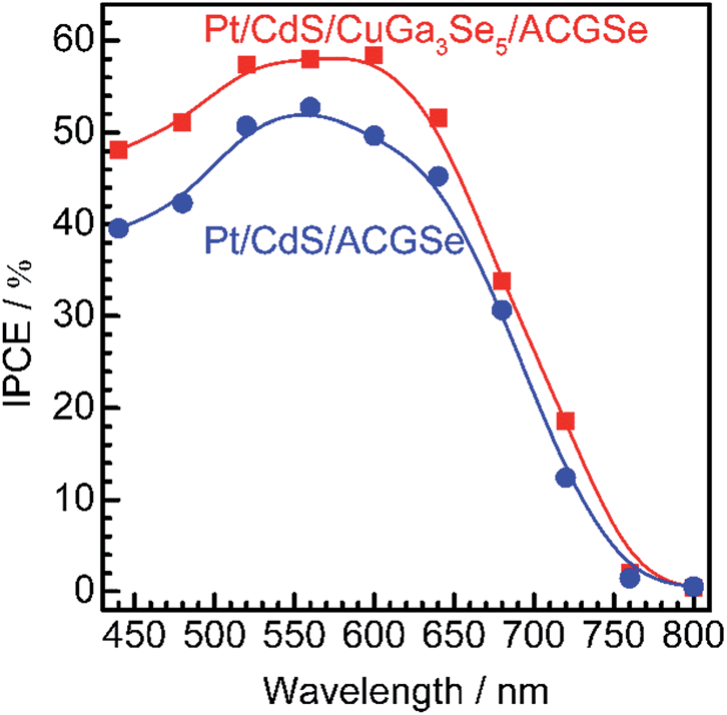
Wavelength dependence of the IPCE spectra for Pt/CdS/CuGa_3_Se_5_/ACGSe with CuGa_3_Se_5_ deposited for 15 min, and Pt/CdS/CGSe measured at an applied potential of 0 V_RHE_. The IPCE was measured in 0.1 M Na_2_HPO_4_ (adjusted to pH 10 by addition of NaOH) under monochromatic light from a 300 W Xe lamp equipped with band-pass filters and an optical fiber.

The measured IPCE spectra of Pt/CdS/CuGa_3_Se_5_/ACGSe and Pt/CdS/CGSe are shown in Fig. 2. Pt/CdS/CuGa_3_Se_5_/ACGSe showed a higher efficiency than Pt/CdS/ACGSe at all measured wavelengths. The IPCE for Pt/CdS/CuGa_3_Se_5_/ACGSe was above 57% in the wavelength range of 520–600 nm. The decrease below 540 nm is thought to be due to photo-absorption and recombination of photo-generated carriers in the CdS layer due to the low crystallinity,^39,46^ which suggests the possibility of further improvement in this region of the spectrum by replacing CdS with a wider-band-gap material such as ZnS.^44^ The IPCE for both Pt/CdS/CuGa_3_Se_5_/ACGSe and Pt/CdS/ACGSe converged to zero at *ca.* 760 nm, suggesting that the ACGSe layers act as photo-absorbers in both photocathodes. The photocurrent under the AM 1.5G spectrum estimated from the IPCE is consistent with the results of the *I*–*E* measurements (see Fig. S4 in ESI†).

**Fig. 3:**
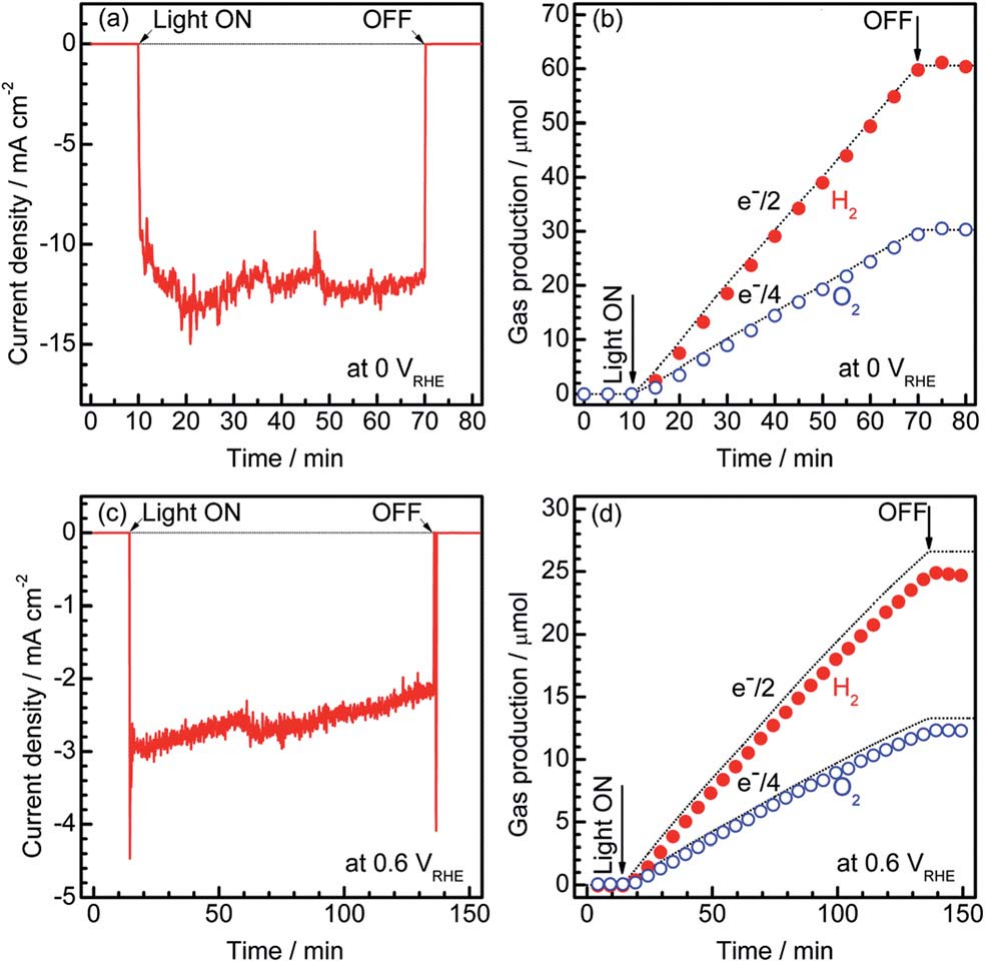
Current–time curves for the Pt/CdS/CuGa_3_Se_5_/ACGSe electrode with CuGa_3_Se_5_ deposited for 15 min at an applied potential of 0 V_RHE_ (a) and 0.6 V_RHE_ (c), and the corresponding amount of H_2_ and O_2_ that evolved during the measurements (b), (d). An airtight 3-electrode configuration was used, with a Pt wire and an Ag/AgCl electrode as the counter and reference electrodes, respectively. A 0.1 M Na_2_HPO_4_ (adjusted to pH 10 by addition of NaOH) and a 150 W Xe lamp (420–800 nm) equipped with a cutoff filter (HOYA, L42) and a cold mirror (Optline, CM-1) were used as the electrolyte and light source, respectively. The dashed lines indicate the expected amounts of oxygen and hydrogen for a Faradaic efficiency of unity.

To estimate the Faradaic efficiency of the Pt/CdS/CuGa_3_Se_5_/ACGSe photocathode for the generation of H_2_ and O_2_, the gas products were analysed using micro-GC (see ESI†). Details of the measurement conditions are described in the ESI.† Fig. 3 shows the current–time curves at different potentials and the corresponding amounts of hydrogen and oxygen evolved on the specimen and Pt counter electrode. Two different applied potentials were used, namely 0 V_RHE_ and 0.6 V_RHE_, and in both cases, stoichiometric hydrogen and oxygen evolution from water was confirmed, as shown in Fig. 3b and d. At 0.6 V_RHE_, a small decrease in the photocurrent was observed after 3 h (see Fig. 3c), likely due to the partial oxidation of CdS, because, at such a high potential, it may not be possible to maintain the entire surface of the CdS layers under reductive conditions.^40^ The hydrogen and oxygen evolution at an applied potential of 0.6 V_RHE_ was slightly lower than that estimated from the photocurrent. This might be due to degradation of the photocathode. The hydrogen evolution at 0.75 V_RHE_ was further confirmed (see Fig. S5 in ESI†), which provides additional evidence for the contribution of the observed small photocurrent at a potential >0.6 V_RHE_ to the hydrogen evolution reaction.

**Fig. 4:**
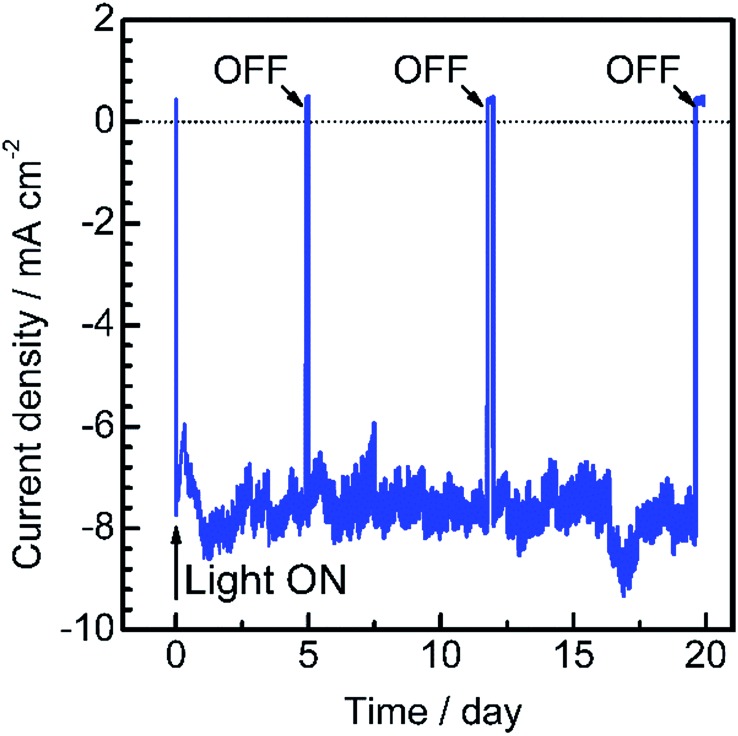
Current–time curve for a Pt/CdS/CuGa_3_Se_5_/ACGSe electrode with CuGa_3_Se_5_ deposited for 15 min at a potential of 0 V_RHE_. A buffer solution of 0.05 M Na_2_HPO_4_ + 0.05 M NaH_2_PO_4_ (adjusted to pH 7 by addition of NaOH) was used as the electrolyte. 420–800 nm light (100 W cm^–2^) from a Xe lamp equipped with a cutoff filter (HOYA, L42) and a cold mirror (Optline, CM-1) was employed as the light source.

To assess the long-term stability of the prepared Pt/CdS/CuGa_3_Se_5_/ACGSe photocathode, the time course of the photocurrent was measured at 0 V_RHE_ in a buffer solution under visible light irradiation, as shown in Fig. 4. After an induction period of a few days, the electrode showed a stable cathodic photocurrent for over 19 days without a detectable decrease. It should be noted that the observed fluctuations in the photocurrent during the measurements were due to the adsorption of bubbles of gas products on the photoelectrode and vigorous magnetic stirring.

### Structural properties of the CuGa_3_Se_5_/ACGSe samples

**Fig. 5:**
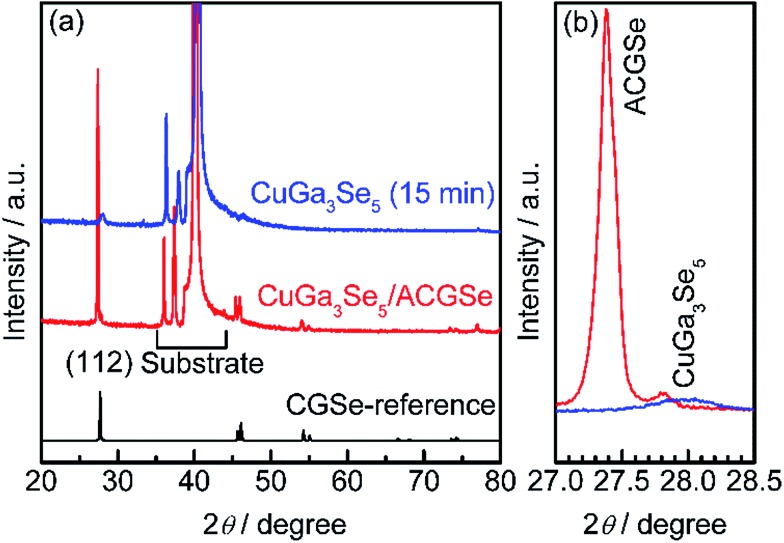
XRD patterns for CuGa_3_Se_5_/ACGSe with CuGa_3_Se_5_ deposited for 15 min, and a reference CuGa_3_Se_5_ film deposited on Mo/Ti/SLG for 15 min (a), and the corresponding (112) diffractions (b). The reference pattern for CGSe (PDF no. 31-456) is shown at the bottom for comparison.

To determine the structural properties of the electrodes, XRD, SEM, and TEM were conducted. XRD patterns for the CuGa_3_Se_5_/ACGSe with CuGa_3_Se_5_ deposited for 15 min and a reference CuGa_3_Se_5_ film deposited on Mo/Ti/SLG for 15 min are shown in Fig. 5. In addition to the markedly strong diffraction peaks associated with the Mo/Ti/SLG substrate, both samples showed a prominent peak at 2*θ* ≈ 27–28°, assignable to the (112) diffraction from a chalcopyrite structure, suggesting that the preferred orientation was (112). The (112) diffraction peak for CuGa_3_Se_5_/ACGSe (Fig. 5b) exhibited a shoulder peak at *ca.* 27.8°, which corresponds to the (112) diffraction peak for CuGa_3_Se_5_.^50,53,54^ This confirmed the presence of the CuGa_3_Se_5_ layer. The differences in the diffraction angle and sharpness of the CuGa_3_Se_5_ (112) peaks for CuGa_3_Se_5_/ACGSe and CuGa_3_Se_5_ are due to intermixing at the CuGa_3_Se_5_/ACGSe interface and the different underlayers.

**Fig. 6:**
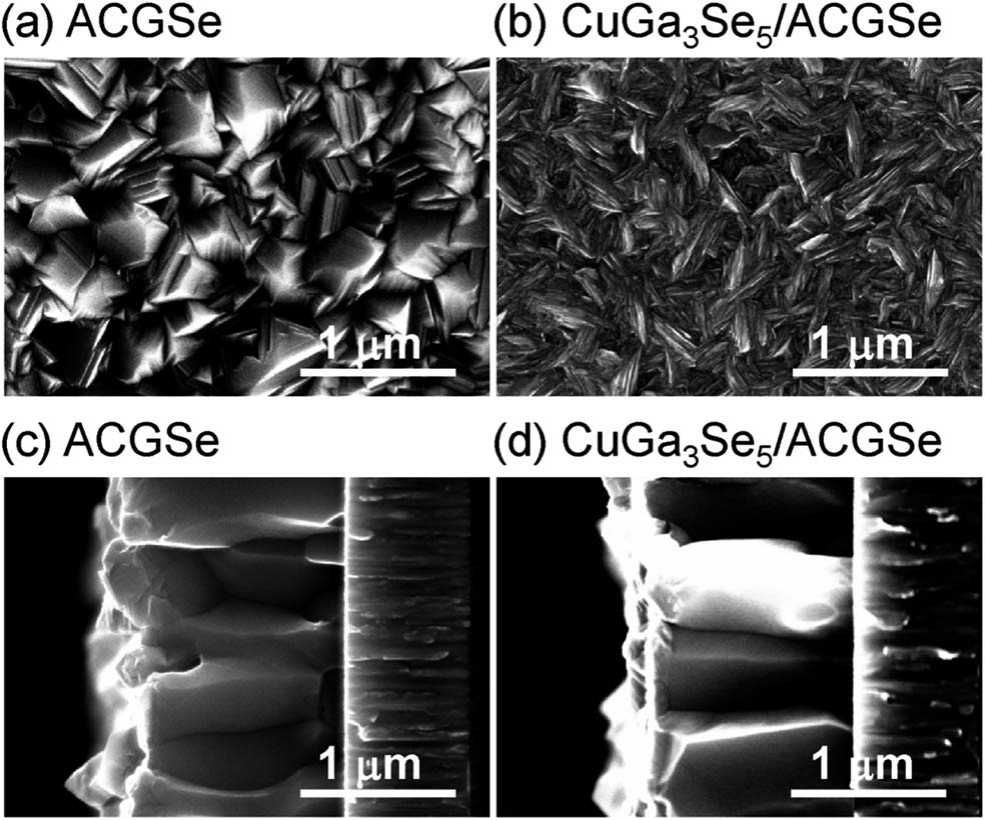
Top and cross-sectional SEM images of ACGSe (a) and (c), and CuGa_3_Se_5_/ACGSe with CuGa_3_Se_5_ deposited for 15 min (b) and (d).

SEM images of the ACGSe and CuGa_3_Se_5_/ACGSe thin films are presented in Fig. 6. The ACGSe showed a rough surface consisting of large polygonal grains (see Fig. 6a), while CuGa_3_Se_5_/ACGSe showed a surface densely covered by flake-shaped grains (Fig. 6b), which is characteristic of CuGa_3_Se_5_ (see Fig. S6 in ESI†).^50,52^ This obvious difference in surface morphology implies that the CuGa_3_Se_5_ layer fully covered the ACGSe underlayer. The cross-sectional SEM image of CuGa_3_Se_5_/ACGSe (Fig. 6d) clearly revealed the compact bilayer structure consisting of a CuGa_3_Se_5_ overlayer and an ACGSe underlayer with columnar grains. The thickness of the CuGa_3_Se_5_ layer was estimated from the SEM image to be about 100 nm, which is less than the thickness estimate of *ca.* 150 nm made on the basis of the deposition rate for the preparation conditions used. This discrepancy is indicative of interdiffusion between the CuGa_3_Se_5_ and ACGSe layers, as revealed by the XRD results. It should be noted that the ACGSe layer in the ACGSe and CuGa_3_Se_5_ samples was composed of large columnar grains, which is consistent with our previous report on ACGSe thin films.^46^ It is well known that large columnar grains are desirable for efficient I–III–VI_2_ thin-film solar cells.^35–37^

**Fig. 7:**
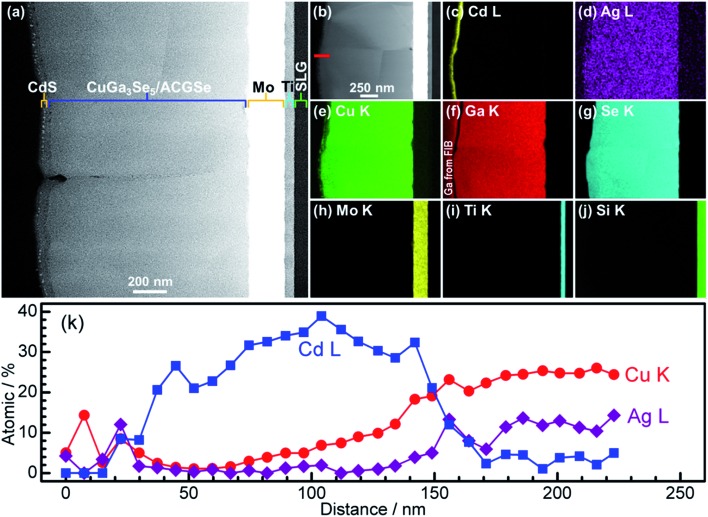
Cross-sectional TEM images of CdS/CuGa_3_Se_5_/ACGSe sample with CuGa_3_Se_5_ deposited for 15 min (a) and (b); EDX elemental maps for Cd (c), Ag (d), Cu (e), Ga (f), Se (g), Mo (h), Ti (i), Si (j); and line profiles for Cd, Cu and Ag across the CdS and CuGa_3_Se_5_/ACGSe interfaces (k). The red line in Fig. 4b indicates where the linear profile was measured. The sample was sliced using a Ga focused ion beam (FIB).

For the CdS-deposited CuGa_3_Se_5_/ACGSe sample, cross-sectional TEM and EDX mapping was conducted to characterize the interfacial structure. The cross-sectional TEM images (Fig. 7a and b) revealed a well-defined layered structure of CdS/CuGa_3_Se_5_/ACGSe/Mo/Ti/SLG, where Mo/Ti/SLG is the substrate. An EDX elemental map of Cd (Fig. 7c) clearly indicated that the CuGa_3_Se_5_/ACGSe surface was fully coated by a CdS layer with a thickness of *ca.* 80 nm. Fig. 7d–g show EDX elemental maps for Ag, Cu, Ga, and Se, and Fig. 7k shows line profiles for Cd, Cu, and Ag across the CdS and CuGa_3_Se_5_/ACGSe interfaces. From Fig. 7k, it can clearly be seen that Cd, Cu and Ag exhibited a concentration gradient, despite the pronounced Cd diffusion.^55^ The concentration gradient for Cu corroborates the presence of a Cu-deficient ODC thin layer.

### Band alignments at solid–liquid interfaces

**Fig. 8:**
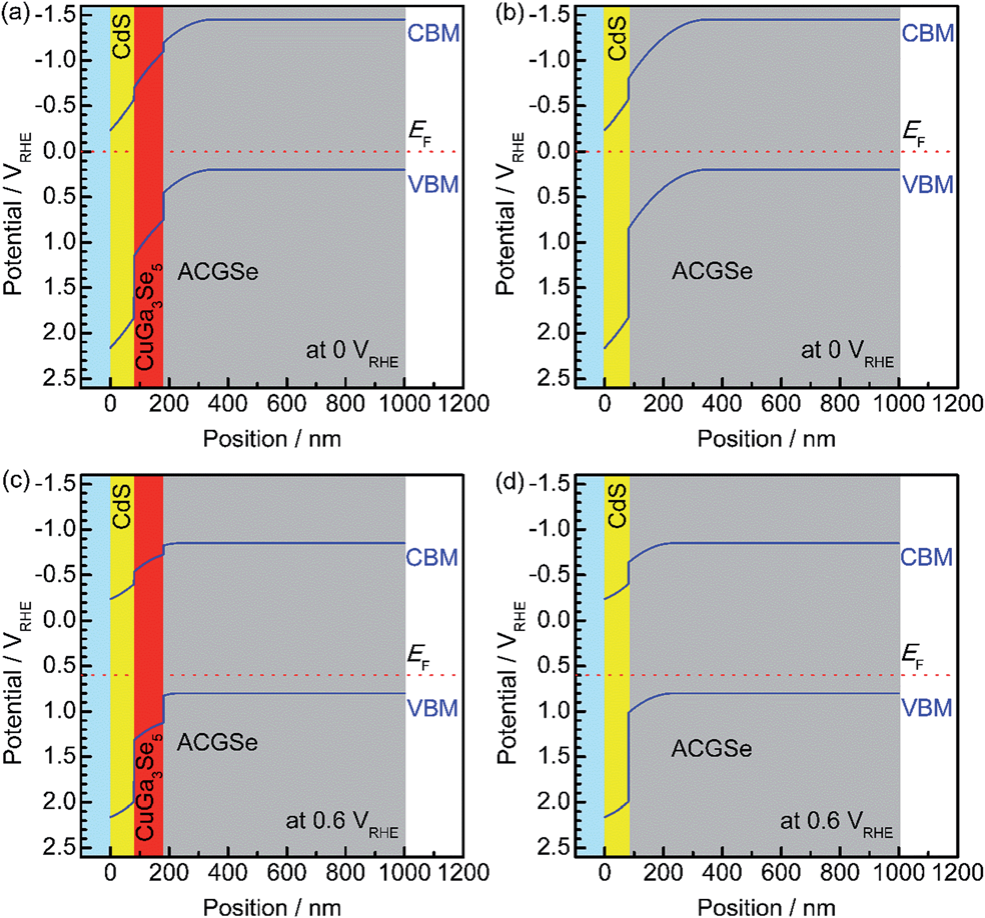
Calculated band alignment near the solid–liquid interface for CdS/CuGa_3_Se_5_/ACGSe with CuGa_3_Se_5_ deposited for 15 min, and Pt/CdS/ACGSe at an applied potential of 0 V_RHE_ (a) and (b), and 0.6 V_RHE_ (c) and (d). VBM, CBM and *E*_F_ denote the valence-band maximum, conduction-band minimum, and Fermi energy, respectively.

To elucidate the beneficial effects of the CuGa_3_Se_5_ layer, the band alignment for the Pt/CdS/CuGa_3_Se_5_/ACGSe and Pt/CdS/ACGSe electrodes at solid–liquid interfaces were calculated numerically by solving the discretized Poisson's equation (see ESI†) as shown in Fig. 8.^40^ For both samples, the width of the depletion layer formed at the solid–liquid interface under applied potentials of 0 V_RHE_ and 0.6 V_RHE_ was estimated to be 348 nm and 234 nm, respectively. Compared with the Pt/CdS/ACGSe electrode (Fig. 8b and d), the CuGa_3_Se_5_-modified sample had a more desirable stepped-down alignment of both the VBM and the conduction-band minimum (CBM) between the CdS and ACGSe layers, as shown in Fig. 8a and c, because the CuGa_3_Se_5_ has a larger band gap and a deeper VBM potential than ACGSe (see Fig. S7–S9 in ESI†).

For the Pt/CdS/ACGSe electrode, photo-generated electron–hole pairs are created mainly within the depletion layer in the ACGSe, and then spatially separated by the built-in electric field, which can be described as band bending in a band diagram. The photo-generated electrons in the conduction band then have to pass through the defect-rich CdS/ACGSe interface, because of the large lattice mismatch and low crystallinity of CdS, in order to evolve hydrogen, while the photo-generated holes in the valence band move to the back contact layer (and finally to the counter electrode *via* the external circuit) for oxygen evolution. Considering this charge separation, it is possible that the CdS/ACGSe interface plays a key role in the recombination of photo-generated carriers.^47,48^ One possible reason for the higher photocurrent and onset potential observed for Pt/CdS/CuGa_3_Se_5_/ACGSe than for Pt/CdS/ACGSe is a reduced amount of recombination at the interfaces. CuGa_3_Se_5_ has almost the same crystal structure as ACGSe, except for the presence of ordered defect complexes and the absence of Ag (partially substituted for Cu in CuGaSe_2_) in the former. In addition, since the CuGa_3_Se_5_ layer is deposited *in situ*, the quality of the CuGa_3_Se_5_/ACGSe interface is likely to be high. Since the CuGa_3_Se_5_ interlayer has a deeper VBM potential than ACGSe, an energy barrier is formed that effectively prevents the diffusion of holes to the defective CdS/CuGa_3_Se_5_ interface, thus suppressing carrier recombination. Note that the cathodic photocurrent at an applied potential of 0.6 V_RHE_ is mainly due to photoexcited electrons generated in the CuGa_3_Se_5_ layer, which is fully depleted and can absorb a portion of the incoming photons with an energy of >1.85 eV. Thus, the photo-excited carriers in the CuGa_3_Se_5_ layers can directly contribute to the photocurrent in Pt/CdS/CuGa_3_Se_5_/ACGSe electrodes because of the presence of a strong built-in electric field and the step-like alignment of both the CBM and VBM.

## Conclusions

CuGa_3_Se_5_-modified ACGSe thin-film photocathodes modified with CdS and Pt were investigated for PEC hydrogen evolution from water under simulated sunlight irradiation. The photocurrent densities and onset potentials for Pt/CdS/CuGa_3_Se_5_/ACGSe photocathodes showed a strong dependence on the thickness of the CuGa_3_Se_5_ layer. The Pt/CdS/CuGa_3_Se_5_/ACGSe photocathode with CuGa_3_Se_5_ deposited for 15 min (*ca.* 100 nm) exhibited a cathodic photocurrent of 8.79 mA cm^–2^ at 0 V_RHE_, an onset potential of 0.62 V_RHE_ under simulated sunlight, and a HC-STH of 1.81% at 0.36 V_RHE_, which were higher than the values of 7.26 mA cm^–2^, 0.48 V_RHE_, and 1.02% for unmodified Pt/CdS/ACGSe. In addition, calculations showed that the inclusion of CuGa_3_Se_5_ led to a more desirable band alignment near the solid–liquid interface because CuGa_3_Se_5_ has a deeper VBM potential than ACGSe. A Pt/CdS/CuGa_3_Se_5_/ACGSe photocathode generated a stable photocurrent for *ca.* 20 days, producing stoichiometric hydrogen from water under visible light irradiation. These results demonstrate the feasibility of using CuGa_3_Se_5_-modified ACGSe electrodes decorated with CdS and Pt as photocathodes in dual-photoelectrode PEC cells for bias-free water splitting, though further improvements including realization of scalable and low cost electrodes by the fabrication methods like the simple particle transfer method are necessary.^25,40^
