# Supplementary material for: Durable hydrogen evolution from water driven by sunlight using (Ag,Cu)GaSe2 photocathodes modified with CdS and CuGa3Se5
Source: Chem Sci. 2014 Sep 4;6(2):894–901. doi: 10.1039/c4sc02346c (PMC5811107; doi:10.1039/c4sc02346c)
Supplement: Supplementary file 1 [file SC-006-C4SC02346C-s001.pdf]

Electronic Supplementary Material (ESI) for Chemical Science.
This journal is © The Royal Society of Chemistry 2014

Supporting Information

Durable Hydrogen Evolution from Water Driven by Sunlight using (Ag, Cu)$GaSe_2$ Photocathodes Modified with CdS and $CuGa_3Se_5$

Li Zhang[a], Tsutomu Minegishi[a], Mamiko Nakabayashi[b], Yohichi Suzuki[c], Kazuhiko Seki[c], Naoya Shibata[b], Jun Kubota[a] and Kazunari Domen[a]*

[a] *Department of Chemical System Engineering, The University of Tokyo, 7-3-1 Hongo, Bunkyo-ku, Tokyo, 113-8656, Japan. Fax: +81-3-5841-8838; Tel: +81-3-5841-1652; E-mail: domen@chemsys.t.u-tokyo.ac.jp*
[b] *Institute of Engineering Innovation, The University of Tokyo, 2-11-16 Yayoi, Bunkyo-ku, Tokyo 113-8656, Japan*
[c] *NRI, National Institute of Advanced Industrial Science and Technology (AIST), AIST Tsukuba Central 5, Higashi 1-1-1, Tsukuba, Ibaraki 305-8565, Japan*

Corresponding Author

* Kazunari Domen

E-mail: domen@chemsys.t.u-tokyo.ac.jp

Supporting Information

Preparation of Mo/Ti/SLG substrates

Mo was deposited onto ultrasonically cleaned soda-lime glass (SLG) substrates as the back contact by radio frequency (RF) magnetron sputtering. Prior to Mo deposition, a thin Ti layer was sputtered as a buffer layer. Sputtering was carried out using high-purity Ti (99.98%) and Mo (99.9%) targets under an Ar atmosphere (8.0×10^{-2} Pa) at an RF power of 100 W. The SLG substrates were kept at ca. 500°C throughout the deposition process, and the deposition times for Ti and Mo were 5 and 20 min, respectively.

Surface modification with CdS

CdS layers were formed on the surface of $CuGa_3Se_5$/ACGSe films by the chemical bath deposition (CBD) method.[1] Prior to CdS deposition, the surface of the $CuGa_3Se_5$/ACGSe films was pretreated with Cd^{2+} by dipping the samples into an aqueous solution containing 2 M NH_4OH (Wako, 28 wt%) and 7.5 mM $Cd(CH_3COO)_2$ (Kanto, 98%) at 80°C for 10 min. CBD of CdS was performed by immersing the Cd^{2+}-pretreated specimens in a bath solution containing 0.375 M $SC(NH_2)_2$ (Kanto, 98%), 7.5 mM $Cd(CH_3COO)_2$, and 2 M NH_4OH at 65°C for 6 min, resulting in the deposition of a CdS layer with a thickness of ca. 80 nm. After CdS deposition, the samples were annealed in air at 300°C for 60 min.

Supporting Information

Surface modification with Pt

The prepared CdS/$CuGa_3Se_5$/ACGSe thin film samples were fabricated into electrodes by connecting a lead wire to the Mo backside layer and covering the unnecessary parts with epoxy resin. The electrodes prepared were then surface-modified with Pt as a hydrogen evolution catalyst by PEC deposition using a 3-electrode setup. PEC deposition of Pt was conducted using a solution containing 10 μM H_2PtCl_6 (Kanto, 98.5%) and 0.1 M Na_2SO_4 (Wako, 99%) at an applied potential of ca. –0.5 to –0.6 $V_{Ag/AgCl}$ under illumination by 420–800 nm light from a 300 W Xe lamp equipped with filters. PEC deposition was continued until the photocurrent saturated. Schematic of photoelectrode fabrication process for the Pt- and CdS-modified $CuGa_3Se_5$/ACGSe thin film electrodes (Pt/CdS/$CuGa_3Se_5$/ACGSe) is illustrated in scheme 1.

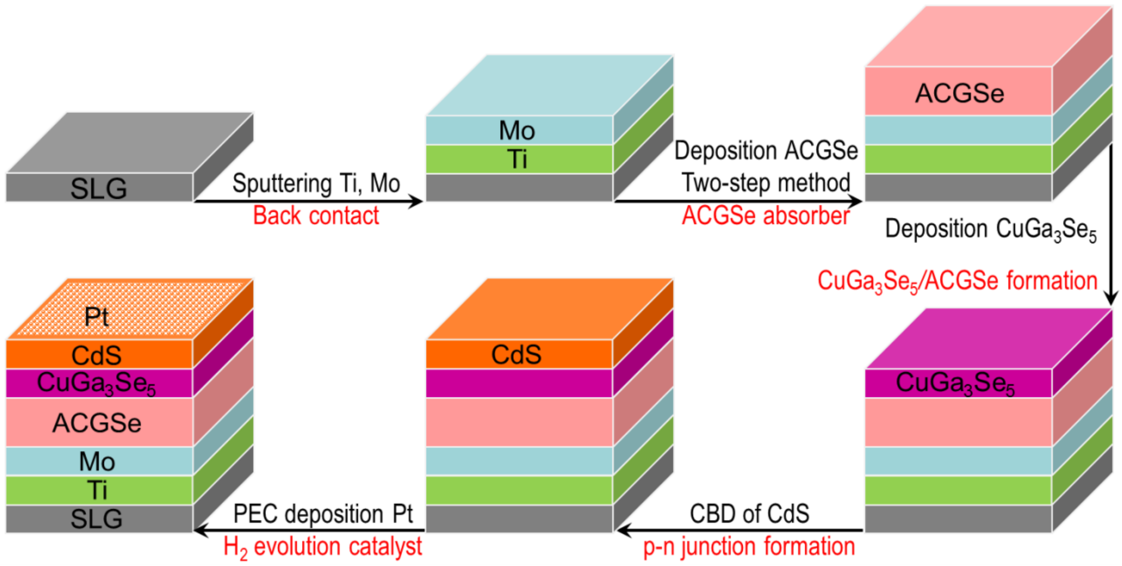

Scheme 1 Schematic of fabrication process for Pt/CdS/$CuGa_3Se_5$/ACGSe electrodes on Mo/Ti/SLG substrates.

Supporting Information

Characterization of ACGSe/$CuGa_3Se_5$ thin films

Structural properties were characterized using X-ray diffraction (XRD; RINT-Ultima III, Rigaku), scanning electron microscopy (SEM; S-4700, Hitachi), and scanning transmission electron microscopy (STEM; JEM-2800, JEOL and EM-002BF-Twin EDS system, JEOL). The crystal structures of the samples were determined using XRD, while the film microstructure was characterized by SEM and TEM. Top and cross-sectional surfaces were studied by SEM. TEM measurements were performed to characterize the cross-sectional structure of the films. The ionization potentials and band gaps of the samples were analyzed by photoelectron spectroscopy in air (PESA; AC-3, Riken Keiki), and UV-vis transmittance spectroscopy (V-670DS, Jasco), respectively.

Gas product analysis

The analysis of gas production was conducted using an airtight 3-electrode cell connected to a gas chromatograph (Agilent 3000 Micro GC, Agilent Technologies). The measurements were performed under an Ar-saturated atmosphere using a 0.1 M aqueous Na_2HPO_4 solution as the electrolyte with the pH adjusted to 10 by NaOH addition. The 420–800 nm light from a 150 W Xe lamp equipped with a cutoff filter (HOYA, L42) and a cold mirror (Optline, CM-1) was employed as light source. The amounts of evolved oxygen and hydrogen were measured by gas chromatography.

Spectrum of the used solar simulator

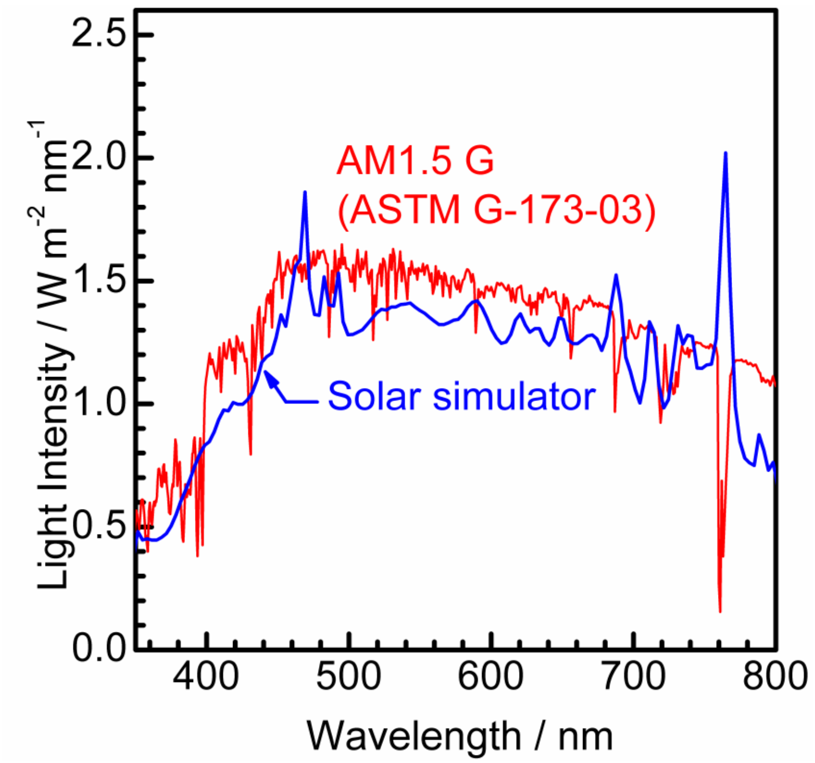

Fig. S1 Spectrum of the used solar simulator in PEC measurements. The spectrum of solar simulator was calibrated to AM 1.5G (ASTM G173-03).

Current-potential (*I*–*E*) curves for Pt/CdS/ACGSe and Pt/CdS/$CuGa_3Se_5$/ACGSe electrodes

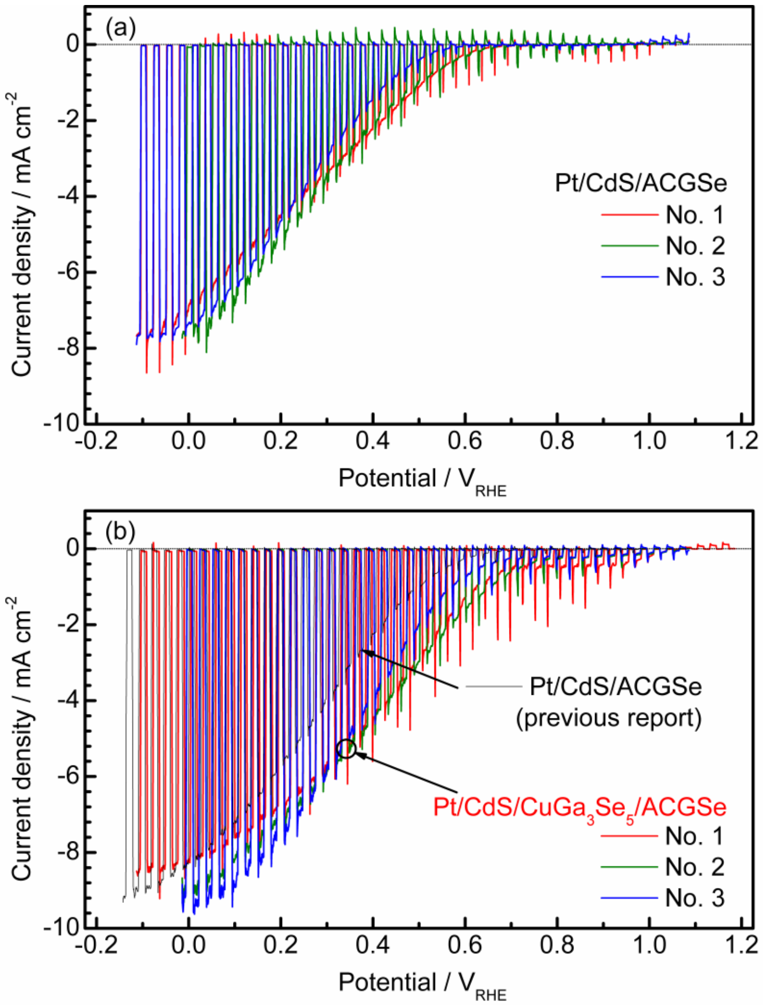

Fig. S2 *I*–*E* curves for Pt/CdS/ACGSe (a), and Pt/CdS/$CuGa_3Se_5$/ACGSe with $CuGa_3Se_5$ deposited for 15 min (b). A 0.1 M aqueous Na_2HPO_4 solution (adjusted to pH 10 by addition of NaOH) was employed as the electrolyte. An applied potential was swept in the positive direction at 5 mV s^{-1} under intermittent irradiation with simulated sunlight. The reported Pt/CdS/ACGSe is shown as a reference, that sample was measured in 0.1 M Na_2SO_4 (adjusted to pH 9.5 by addition of NaOH) under simulated sunlight irradiation.[2]

Half-cell solar-to-hydrogen efficiency (HC-STH) for Pt/CdS/$CuGa_3Se_5$/ACGSe electrode

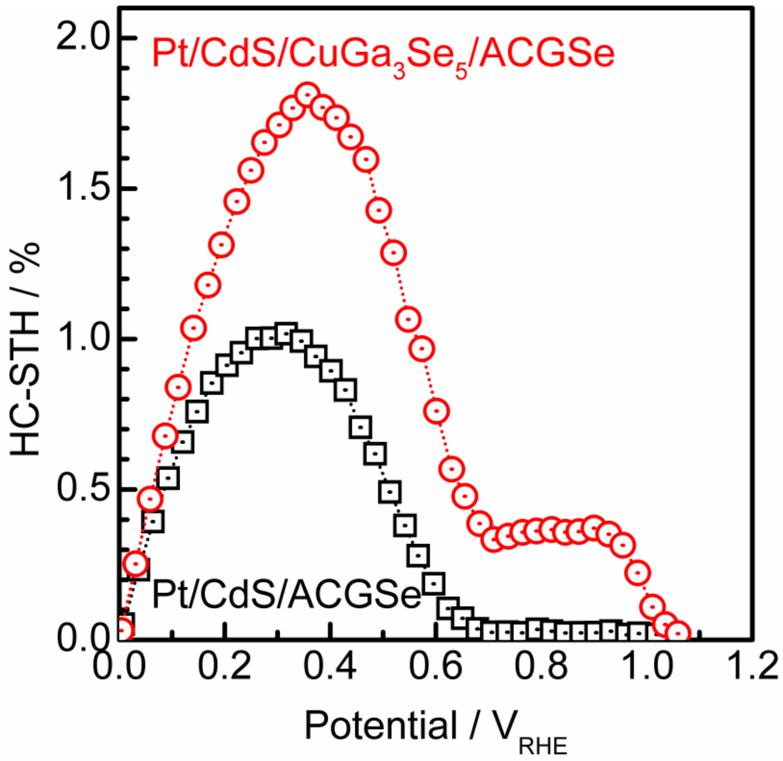

Fig. S3 HC-STH values for the Pt/CdS/ACGSe and Pt/CdS/$CuGa_3Se_5$/ACGSe calculated from the *I*–*E* curves under the simulated sunlight shown in Fig. 1(c).

Calculated photocurrent for Pt/CdS/$CuGa_3Se_5$/ACGSe electrode

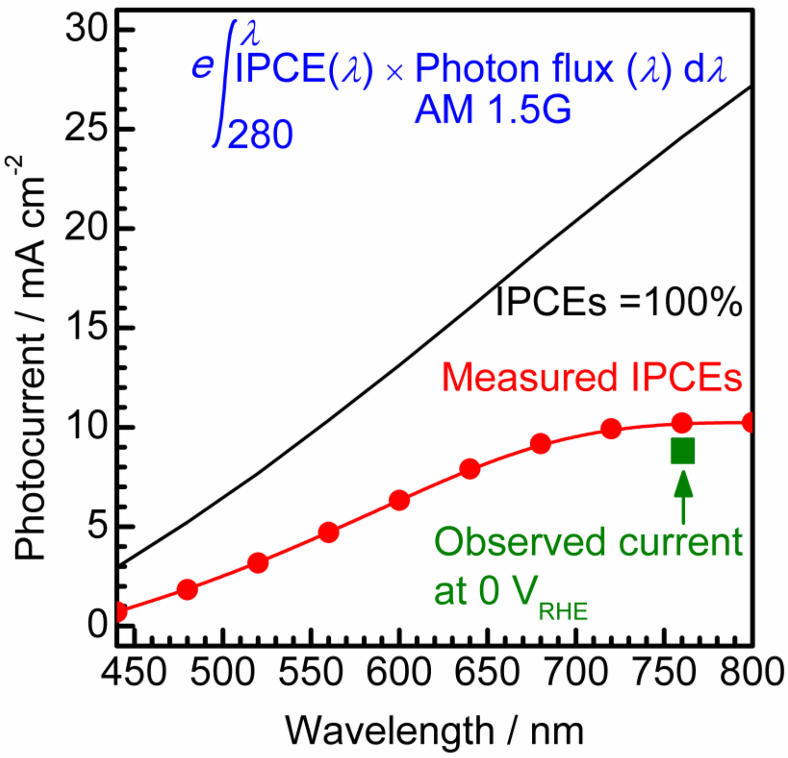

Fig. S4 Calculated photocurrent for Pt/CdS/$CuGa_3Se_5$/ACGSe with $CuGa_3Se_5$ deposited for 15 min. The calculation was done by integrating the measured IPCE for Pt/CdS/$CuGa_3Se_5$/ACGSe over the AM 1.5G spectrum (ASTM G173-03). A photocurrent of 10.2 mA cm^{-2} was calculated at a wavelength of 760 nm, which is only slightly larger than the photocurrent of ca. 8.8 mA cm^{-2} at 0 V_{RHE} determined from the *I*–*E* curves, indicating the accuracy of the measured *I*–*E* curves and IPCE spectra. In addition, the discrepancy between the observed photocurrent and the theoretical maximum photocurrent of 24 mA cm^{-2} at 760 nm, obtained by assuming 100% IPCE, indicates a high potential for further improvement.

Gas product analysis for Pt/CdS/$CuGa_3Se_5$/ACGSe at high applied potential

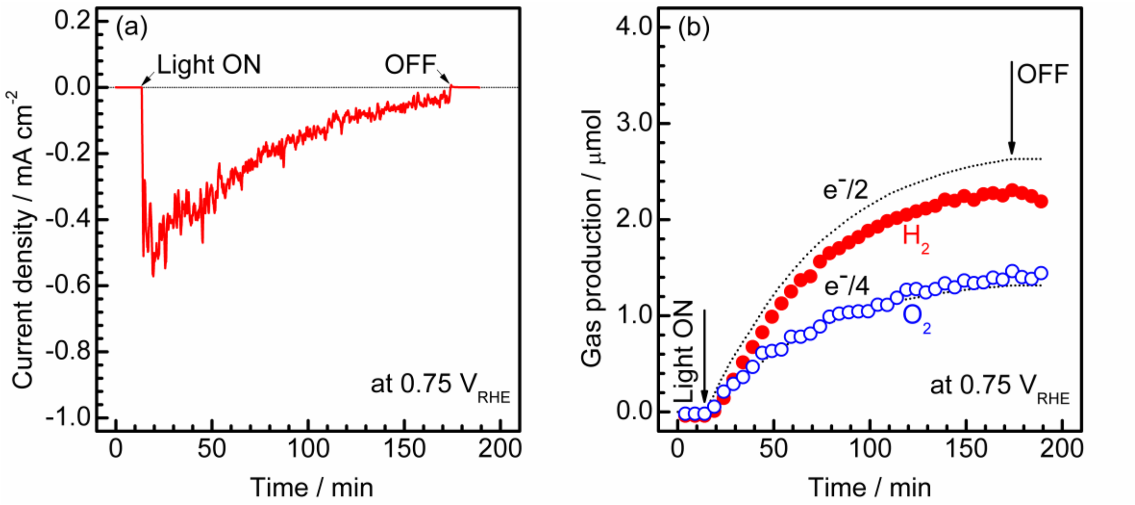

Fig. S5 Current-time curve for the Pt/CdS/$CuGa_3Se_5$/ACGSe electrode with $CuGa_3Se_5$ deposited for 15 min at an applied potential of 0.75 V_{RHE} (a), and the corresponding amount of H_2 and O_2 that evolved during the measurement (b). An airtight 3-electrode configuration was used, with a Pt wire and an Ag/AgCl electrode as the counter and reference electrodes, respectively. A 0.1 M Na_2HPO_4 (adjusted to pH 10 by addition of NaOH) and a 150 W Xe lamp (420–800 nm) equipped with a cutoff filter (HOYA, L42) and a cold mirror (Optline, CM-1) were used as the electrolyte and light source, respectively. The dashed lines indicate the expected amounts of hydrogen and oxygen for a Faradaic efficiency of unity.

Surface morphology of $CuGa_3Se_5$ reference sample

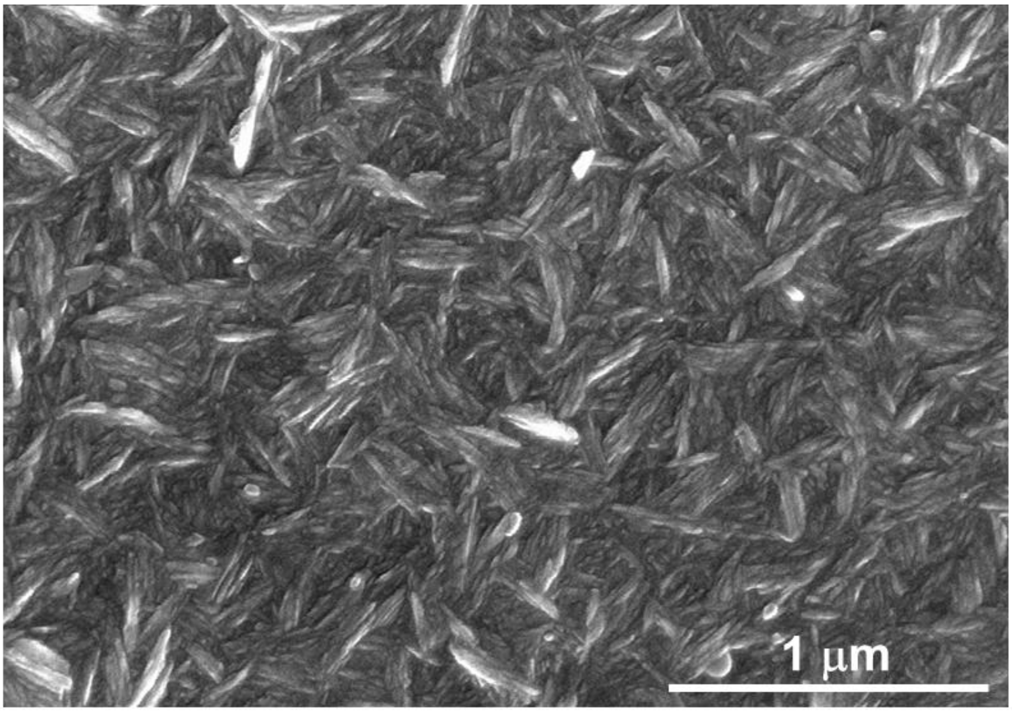

Fig. S6 Top-view SEM image of $CuGa_3Se_5$ reference sample deposited for 15 min on Mo/Ti/SLG substrate.

Band gaps and VBM potentials for ACGSe and $CuGa_3Se_5$ reference samples

The band gap and VBM potential for the ACGSe and $CuGa_3Se_5$ reference samples were investigated using UV-vis transmittance and photoelectron spectroscopy in air (PESA), respectively. UV-vis transmittance spectra of ACGSe and $CuGa_3Se_5$ are shown in Fig. S7. The band gap for ACGSe and $CuGa_3Se_5$ was estimated to be ca. 1.65 and 1.85 eV (Fig. S8), respectively, using the Tauc method.[3]

PESA spectra of the ACGSe and $CuGa_3Se_5$ reference samples are shown in Fig. S9. The VBM potential vs. NHE for $CuGa_3Se_5$ was ca. 0.25 V higher than that for ACGSe. On the basis of the variations in the band gap and the VBM potential, we concluded that the increase in the band gap for $CuGa_3Se_5$ was mainly due to deepening of the VBM position.

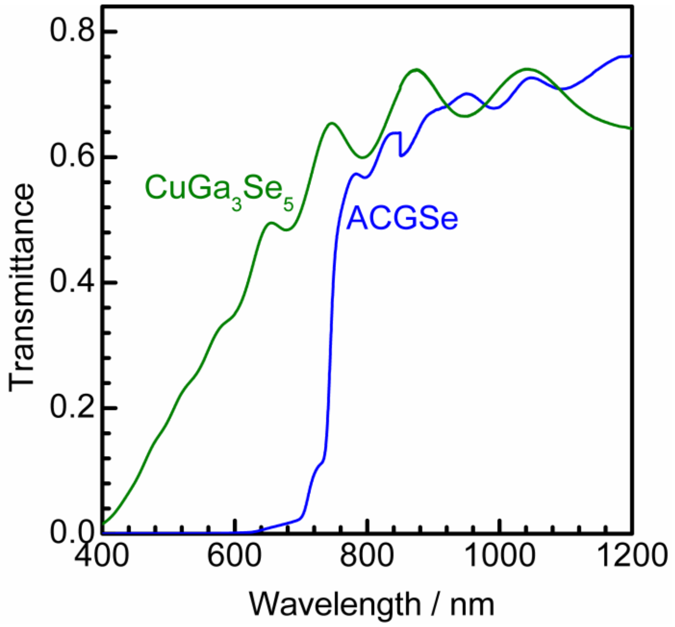

Fig. S7 UV-vis transmittance spectra of the ACGSe and $CuGa_3Se_5$ (15 min) reference samples deposited on FTO substrates.

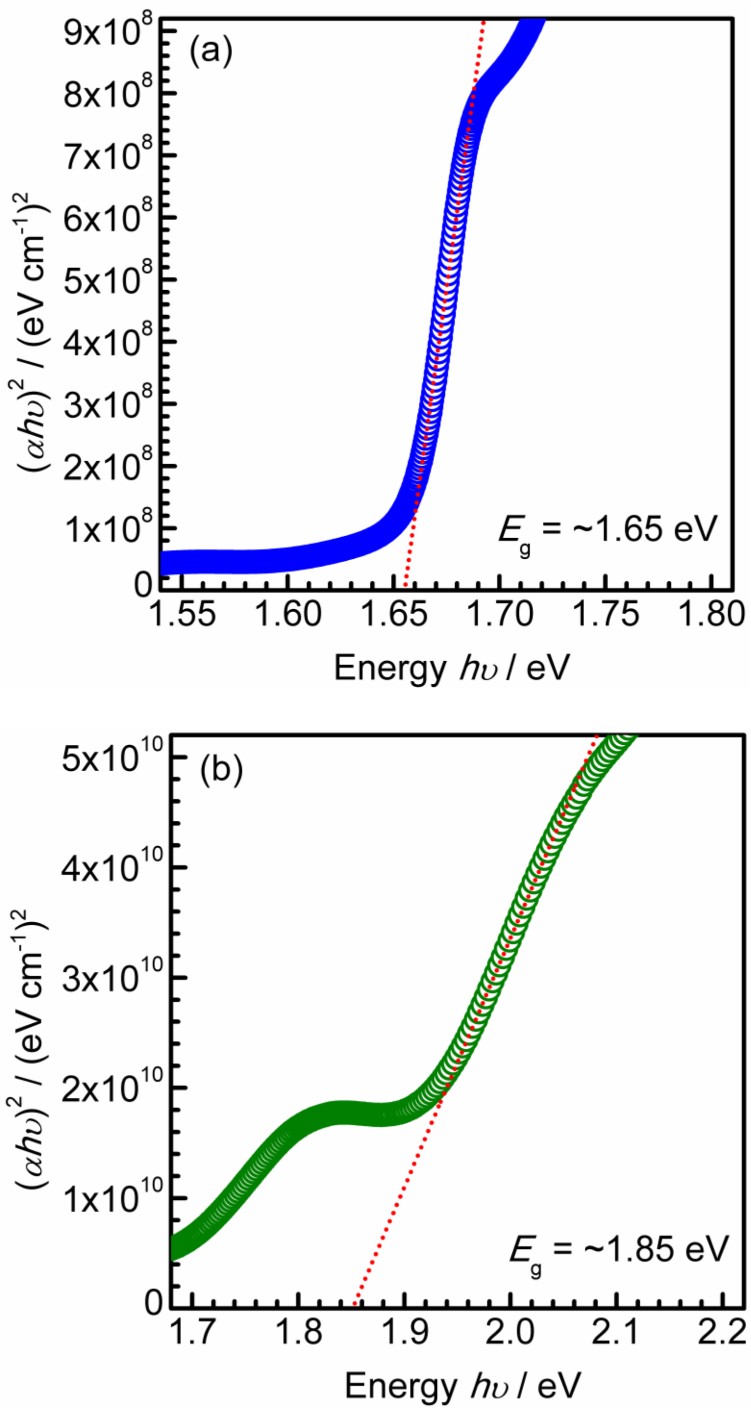

Fig. S8 Tauc plots for the reference samples of ACGSe (a), and $CuGa_3Se_5$ (b). The band gap was determined by using the Tauc extrapolation method.[3]

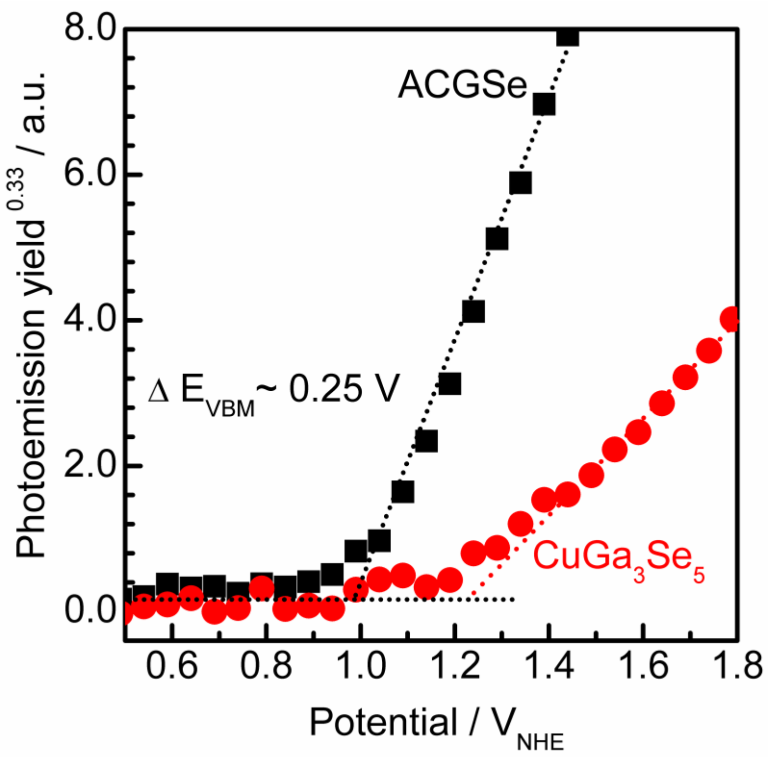

Fig. S9 PESA spectra of the ACGSe and $CuGa_3Se_5$ (15 min) reference samples prepared on Mo/Ti/SLG substrates. The valence band maximum (VBM) potential for $CuGa_3Se_5$ was ca. 0.25 V higher than that for ACGSe.

Band alignment at solid−liquid interface

The band alignment at the solid−liquid interface was calculated by solving Poisson's equation, which for CdS and ACGSe (or $CuGa_3Se_5$), is respectively given by

$$\frac{d^2\varphi}{dx^2} = -\frac{qN_D}{\varepsilon\varepsilon_0} \quad (S1)$$

$$\frac{d^2\varphi}{dx^2} = \frac{qN_A}{\varepsilon\varepsilon_0} \quad (S2)$$

where φ, x, q and ε_0 are the built-in potential, position, electron charge (1.602×10^{-19} C), and vacuum permittivity (8.85×10^{-12} F m^{-1}), respectively. The relative dielectric constant (ε) of CdS and ACGSe was assumed to be 10.[4,5] The concentration of donors (N_D) in the CdS layer and acceptors (N_A) in the ACGSe (or $CuGa_3Se_5$) layer was set to 10^{16}.[4,5] The band gap for ACGSe, $CuGa_3Se_5$, and CdS was 1.65 (see Fig. S8(a)),[2] 1.85 (see Fig. S8(b))[6] and 2.4 eV,[7] respectively. The parameters used for the calculation of the band alignments are shown in Table S1.

The difference between the Fermi level (E_F) and the CBM for CdS, and that between E_F and the VBM for ACGSe (or $CuGa_3Se_5$), was assumed to be 0.2 eV.[4,5] The VBM offset at the CdS/ACGSe and $CuGa_3Se_5$/ACGSe interfaces was 0.98[8] and 0.3 eV (see Fig. S9),[9] respectively. Based on these values and the transitivity rule,[10] the VBM offset at the CdS/$CuGa_3Se_5$ interface was determined to be 0.68 eV. The flat-band potential for CdS was set to −0.04 V_{RHE} for pH 10 derived from −0.1 V_{RHE} (pH 9).[4,5,11]

Supporting Information

Table S1 Semiconductor parameters used to calculate the band alignments.

Parameters	CdS	$CuGa_3Se_5$	ACGSe
Thickness t (nm)	80	100	>1000
Donor density N_D (cm^{-3})	1.0×10^{16}	–	–
Acceptor density N_A (cm^{-3})	–	1.0×10^{16}	1.0×10^{16}
Band gap E_g (eV)	2.4	1.85	1.65
Relative dielectric constant ε	10	10	10

1. M. A. Contreras, M. J. Romero, B. To, F. Hasoon, R. Noufi, S. Ward and K.Ramanathan, *Thin Solid Films*, 2002, **403–404**, 204–211.
2. L. Zhang, T. Minegishi, J. Kubota and K. Domen, *Phys. Chem. Chem. Phys.*, 2014, **16**, 6167–6174.
3. J. Tauc and A. Menth, *J. Non-Cryst. Solids*, 1972, **8–10**, 569–585.
4. M. Moriya, T. Minegishi, H. Kumagai, M. Katayama, J. Kubota and K. Domen, *J. Am. Chem. Soc.*, 2013, **135**, 3733–3735.
5. H. Kumagai, T. Minegishi, Y. Moriya, J. Kubota and K. Domen, *J. Phys. Chem. C*, 2014, **118**, 16386–16392.
6. G. Orsal, F. Mailly, N. Romain, M.C. Artaud, S. Rushworth and S. Duchemin, *Thin Solid Films*, 2000, **361–362**, 135–139.
7. H. Khallaf, I. O. Oladeji, G. Chai and L. Chow, *Thin Solid Films*, 2008, **516**, 7306–7312.
8. T. Schulmeyer, R. Kniese, R.Hunger, W. Jaegermann, M. Powalla and A. Klein,

Thin Solid Films, 2004, **451–452**, 420–423.

9. M. Bär, M. Rusu, S. Lehmann, T. Schedel-Niedrig and I. Lauermann, *Appl. Phys. Lett.*, 2008, **93**, 232104.
10. D. Schmid, M. Ruckh, F. Grunwald and H. W. Schock, *J. Appl. Phys.*, 1993, **73**, 2902–2909.
11. T. Watanabe, A. Fujishima and K. I. Honda, *Chem. Lett.*, 1974, **3**, 897–900.
